# Spatiotemporal variations and determinants of overweight/obesity among women of reproductive age in urban India during 2005-2021

**DOI:** 10.1186/s12889-023-16842-x

**Published:** 2023-10-05

**Authors:** Aditya Singh, Subhojit Let, Seema Tiwari, Mahashweta Chakrabarty

**Affiliations:** 1 Department of Geography, Banaras Hindu University, Varanasi, Uttar Pradesh India; 2 Geography Section, Mahila Maha Vidyalaya, Banaras Hindu University, Varanasi, Uttar Pradesh India; 3 Girl Innovation, Research, and Learning (GIRL) Center, Population Council, New York, USA

**Keywords:** Overweight, Obesity, Obese, NFHS, Reproductive Age, BMI, Women’s Health, India, Urban

## Abstract

**Background:**

India has witnessed rapid urbanization in recent decades, leading to a worrisome surge in non-communicable diseases, particularly overweight/obesity, which now present a critical public health concern. Therefore, this study seeks to examine spatiotemporal variations and determinants of overweight/obesity among women of reproductive age (WRA) in urban India and its states during 2005-2021.

**Methods:**

The study used 44,882, 171,443, and 135,272 WRA aged 15–49 from National Family Health Survey (NFHS)-3 (2005-06), NFHS-4 (2015-16), and NFHS-5 (2019-21), respectively. The outcome variable was overweight/obesity, defined as a Body Mass Index (BMI) of ≥ 25 kg/m^2^. Chi-squared test and multivariable logistic regression were used to identify the determinants of overweight/obesity.

**Results:**

Overweight/obesity prevalence among WRA in urban India has risen significantly, from 23% in 2005-06 to 33% in 2019-21. This increase is particularly pronounced among SC/ST women and women with lower educational levels. During the study period, overweight/obesity rates in different states exhibited varying increases, ranging from 3 percentage points (pp) in Rajasthan to 22 pp in Odisha. Certain southern (e.g., Tamil Nadu and Andhra Pradesh) and northeastern states saw a significant 15 pp or more increase. In contrast, several northern, central, and eastern states (e.g., Punjab, Haryana, Rajasthan, Madhya Pradesh, Chhattisgarh, Jharkhand, West Bengal) experienced relatively smaller increases ranging from 5 to 8 pp. As of 2019-21, two regions exhibited high prevalence rates of overweight/obesity, exceeding 35%: the southern region (Tamil Nadu, Andhra Pradesh, Kerala, and Karnataka) and the northern region (Punjab, Himachal Pradesh, Uttarakhand, and Haryana). In contrast, the Empowered Action Group states had relatively lower rates (25% or less) of overweight/obesity. Regression results showed that older women [AOR: 5.98, 95% CI: 5.71–6.27], those from the richest quintile [AOR: 4.23, 95% CI: 3.95–4.54], those living in south India [AOR: 1.77, 95% CI: 1.72–1.82], and those having diabetes [AOR: 1.92, 95% CI: 1.83–2.02] were more likely to be overweight/obese.

**Conclusion:**

Considering the significant increase in overweight/obesity among urban WRA in India, along with substantial disparities across states and socioeconomic groups, it is imperative for the government to formulate state-specific strategies and policies based on determinants to effectively combat overweight/obesity.

**Supplementary Information:**

The online version contains supplementary material available at 10.1186/s12889-023-16842-x.

## Background

Overweight/obesity, defined as abnormal or excessive fat accumulation that may impair health, has emerged as a significant global health challenge in recent years [1]. Between 1975 and 2016, overweight/obesity rates saw a threefold increase worldwide [1]. Globally, 39% of adults aged 18 years and above were overweight/obese in 2016 [1]. Forecast suggests that by 2030, the population of overweight/obese adults might reach a staggering 1 billion [2]. India, the world’s most populous country, follows this global trend [3]. By 2030, it is projected that around 64 million individuals in India will be living with obesity alone, placing the country 3rd in rank after the United States and China [2]. The impact of overweight/obesity is substantial. It has emerged as an epidemic worldwide, with 2.8 million people dying yearly due to this condition [4]. Failing to address the issue not only jeopardizes the efforts to achieve Sustainable Development Goals (SDGs), but also brings a significant economic and societal repercussions [2, 5]. It is estimated that if left unchecked, overweight/obesity related medical costs in India will reach up to $479 billion by 2060 [2].

While urban areas often act as the engines of a nation’s economic prosperity and provide advanced healthcare facilities, previous evidence suggests that burden of non-communicable diseases (NCDs), including overweight/obesity is much higher in the urban area than in rural areas [6–9]. Existing research attributes this paradoxical phenomenon to several key factors, including the shifting dietary preferences, altered physical activity patterns, and evolving lifestyle choices among the urban population, all of which collectively contribute to an increased susceptibility to overweight/obesity [7, 10, 11]. It is important to highlight that the swift rise in the burden of overweight/obesity within urban areas is generally far from consistent. Instead, it exhibits significant variations among urban populations across various dimensions, including socioeconomic status, biodemographic factors, and geographic locations [12–16]. With India hosting the world’s second-largest urban population (approximately 500 million) and experiencing rapid urbanization characterized by the significant growth of urban centers and the presence of some of the world’s largest metropolitans, there is a heightened need to direct attention towards the burden of overweight/obesity among its urban population [17, 18].

Overweight/obesity is usually more prevalent among women than men [1, 19]. Within women, the women of reproductive age (WRA) emerge as a particularly crucial population to study. WRA’s health not only matters for their own well-being but profoundly influences future generations [20]. Overweight/obesity among WRA is linked to higher risks of gestational diabetes, pre-eclampsia, birthing complications, and maternal/infant mortality [12, 21, 22]. Furthermore, evidence suggests that this group exhibits an elevated vulnerability to a range of NCDs, encompassing cardiovascular disease, diabetes, hypertension, and diverse cancers, thereby contributing to the overall burden of NCD-related morbidity and mortality [1, 13, 23–25]. Addressing the overweight/obesity burden among this subgroup of the population is imperative to reduce NCD-related morbidity and mortality, thereby safeguarding the pursuit of healthy lives for all, as outlined in SDG-3 [26].

The research landscape in India currently has few studies examining different aspects of overweight/obesity, particularly among WRA in urban settings over time [7, 8, 11, 27–29]. Earlier research has attempted to identify the determinants of overweight/obesity among WRA, and consistently indicated that the proportion of overweight/obesity grows with women’s age, level of education, urbanization, household wealth and parity [7, 8, 14, 30]. Furthermore, some studies have indicated that excessive consumption of alcohol, cigarette smoking, and sedentary lifestyle habits are also associated with overweight/obesity among WRA [12, 31]. It’s worth noting that while these studies have recognized a growing prevalence of overweight/obesity in urban India, they are relatively dated and lack inclusion of the most recent data, creating a significant void in our understanding of current trends and patterns of overweight/obesity among WRA in urban India.

Addressing this critical void, our study undertakes a comprehensive analysis of the trends of overweight/obesity among WRA in urban India. Our investigation spans a significant 15-year period from 2005 to 2021, with a specific emphasis on understanding state-level variations in prevalence of overweight/obesity over this time frame. Additionally, we attempt to understand the determinants of overweight/obesity in this demographic. We believe our findings will provide actionable insights for policymakers to combat overweight/obesity and enhance people’s health.

## Methods

### Data source

The study utilizes data from the National Family Health Survey (NFHS)-3, 4, and 5, conducted during 2005-06, 2015-16, and 2019-21, respectively. NFHS, India’s Demographic and Health Survey (DHS), is a large-scale, multi-round survey designed to collect data concerning a wide array of indicators associated with reproductive health, maternal, newborn, and child health, healthcare utilization, maternal and infant nutrition, substance abuse, domestic violence, menstrual hygiene, and various other interconnected domains. Using a two-stage stratified random sampling in all rounds, NFHS-3, NFHS-4 and NFHS-5 interviewed 124,385, 699,686 and 724,115 women aged 15–49 years, respectively. Notably, the response rates were high, with NFHS-3 achieving a rate of 98%, while NFHS-4 and NFHS-5 maintained response rates of 97% each [32–34].

### Study samples

**Fig. 1:**
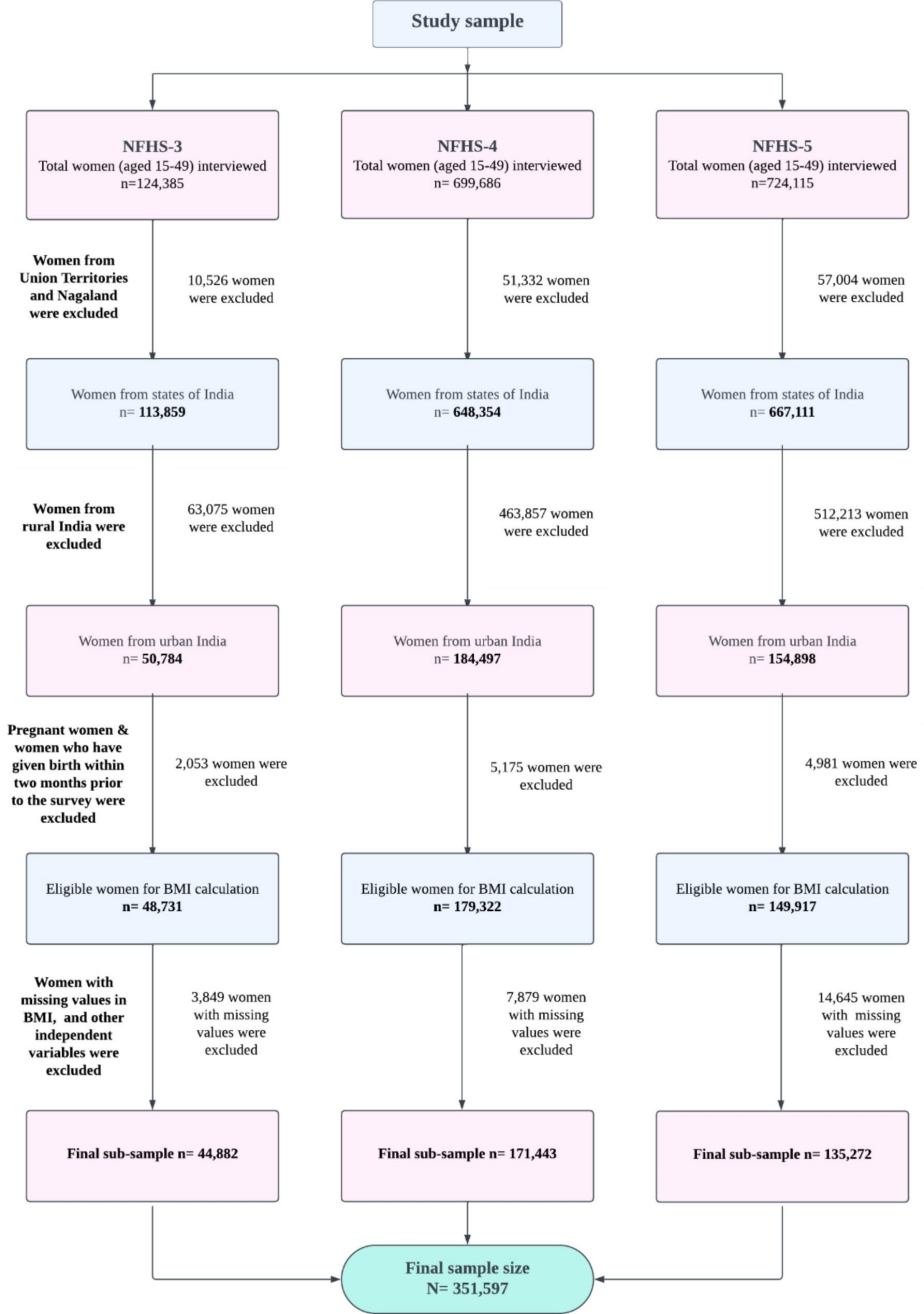
Flowchart showing the selection process of the study sample

For the present study, data was extracted from three consecutive rounds of the NFHS. A total of 44,882, 171,443, and 135,272 sampled women (aged 15–49), who were non-pregnant during the survey and had not given birth within the last two months, were chosen from NFHS-3, NFHS-4, and NFHS-5 datasets, respectively. The detailed process of sample selection for this study is given in Fig. 1.

In NFHS-3, there was a state named Andhra Pradesh, which was subsequently divided into Telangana and Andhra Pradesh in 2014. To maintain consistency in our findings, we combined the state of Telangana with Andhra Pradesh in our analysis for NFHS-4 and NFHS-5. This approach allowed us to ensure that our results were comparable across different survey periods. It is important to note that the state of Nagaland did not have anaemia observations in NFHS-3. Since anaemia is an important variable in our analysis, we opted to remove the observations of Nagaland from all NFHS datasets to maintain the consistency of the analysis.

Furthermore, we decided not to include data from Union Territories (UTs) in our analysis due to the lack of availability of data on UTs in NFHS-3. In addition, in subsequent NFHS rounds (NFHS-4 and NFHS-5), there were changes in the number of UTs, and certain states underwent transformation into new UTs. For example, Jammu and Kashmir were divided into two separate UTs, Jammu & Kashmir and Ladakh during NFHS-5. These administrative divisions posed challenges in the inclusion of UTs in our analysis, and hence, we chose to exclude UTs from the study. It is important to mention that in this study, the terms “women of reproductive age” (WRA) and “women” were used interchangeably.

### Conceptual framework

**Fig. 2:**
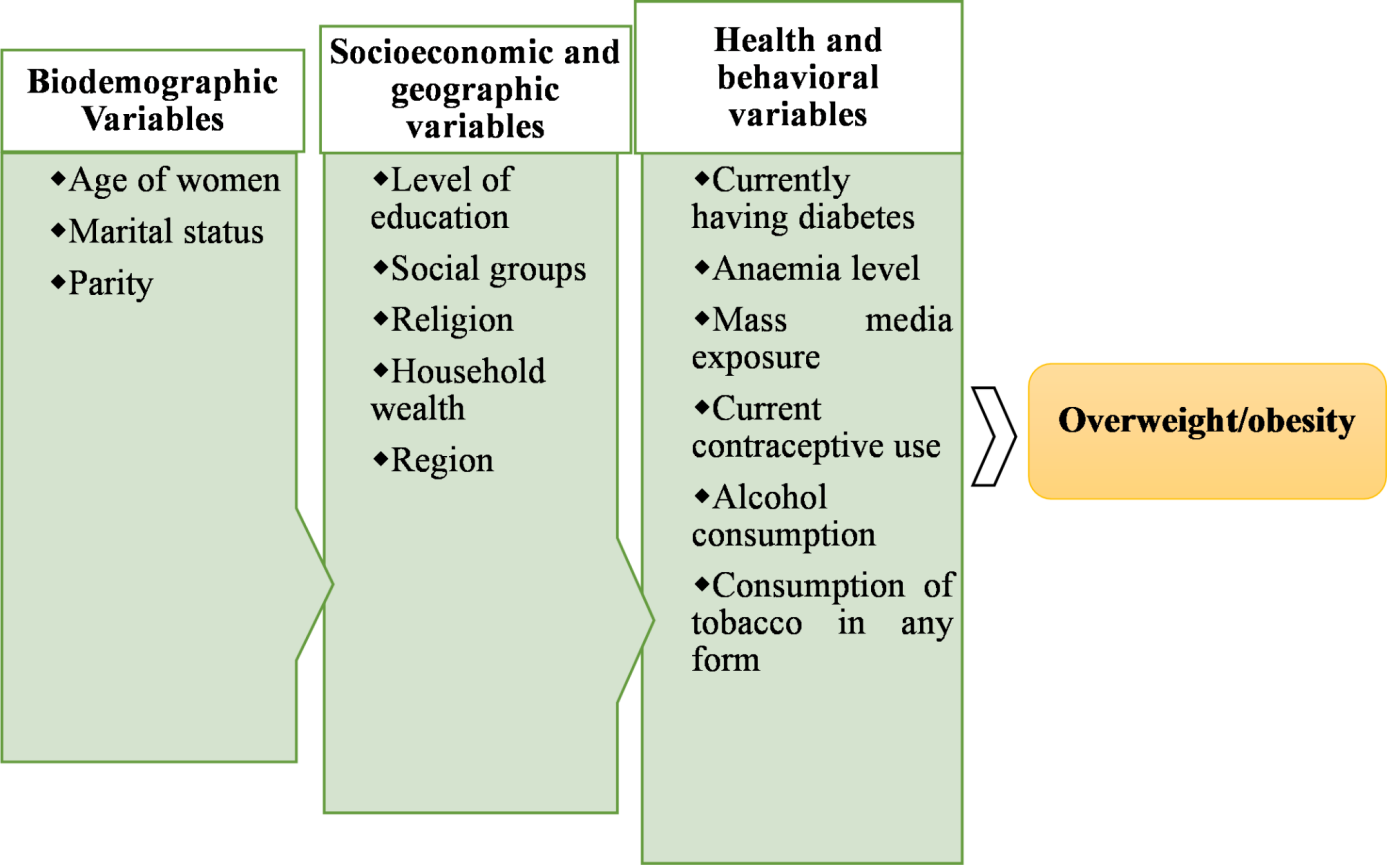
Conceptual framework showing determinants of overweight/obesity among WRA

The present study employs a conceptual framework adapted from the existing literature on overweight/obesity [6, 7, 9, 13, 35, 36]. This framework outlines various variables associated with overweight/obesity, which may influence its prevalence in urban India. The framework identifies three principal domains of variables (biodemographic, socioeconomic and geographic, health and behavioral) which are explained in subsequent sections. This framework guides the study analysis and is visually represented in Fig. 2.

### Dependent variable

The World Health Organization (WHO) defines overweight/obesity as having a Body Mass Index (BMI) of ≥ 25.0 kg/m² [37]. For our study, we categorized women into two groups according to their BMI: those with a BMI of 25 kg/m² or higher were coded as ‘1', indicating ‘overweight/obese’; while those with a BMI below 25 kg/m² were coded as ‘0', indicating ‘not overweight/obese’.

### Independent variables

In this study, a set of independent variables was used to explore the determinants of overweight/obesity among WRA. We reviewed the previous literature on this issue and identified a number of potential variables to be included in the analysis [7, 8, 11, 28, 29, 38, 39]. These variables included age, marital status, parity, level of education, social groups, religion, household wealth, region, mass media exposure, currently having diabetes, anaemia level, current contraceptive use, alcohol consumption, and tobacco consumption in any form. We classified these variables under three major domains: (a) biodemographic, (b) socioeconomic and geographic, and (c) health and behavioral variables. Table 1 contains a comprehensive description of these variables.

**Table 1:** Description of independent variables

Independent variables	Description (with codes)
***Biodemographic variables***	
Age (in years)	In the NFHS, women were asked how old they were on their last birthday, which tells us their current age. We divided the women into four age groups: those who were ‘15–19’ years old (coded as 1), ‘20–29’ years old (coded as 2), ‘30–39’ years old (coded as 3), and ‘40–49’ years old (coded as 4).
Marital status	In the NFHS, women were asked about their current marital status. The options were: “never in union”, “married”, “living with a partner”, “widowed”, “divorced”, or “no longer living together/separated”. We categorized their marital status into three groups: ‘Currently married’ (coded as 1) if the woman was presently married and living with her partner. ‘Not married’ (coded as 2) if the woman had never been in a union. ‘Formerly married’ (coded as 3) if the woman was widowed, divorced, or no longer living together/separated.
Parity	Parity is a term used to indicate how many live births a woman has had up to a specific point in time. We categorized this variable into four groups: ‘No children’ (coded as 0) if the woman had not given birth. ‘1–2 children’ (coded as 1) if the woman had one or two children. ‘3–4 children’ (coded as 2) if the woman had three or four children. ‘5 and above’ (coded as 3) if the woman had five or more children.
***Socioeconomic and geographic variables***	
Level of education	The women were initially asked whether they had attended school. If they responded positively, they were then queried about the number of years they had spent in school. This information was then used to classify their educational level into four categories: ‘no education’ (coded as 0), ‘primary’ (coded as 1, encompassing those with 1 to 5 years of schooling), ‘secondary’ (coded as 2, covering those with 6 to 12 years of schooling), and ‘higher’ (coded as 3, including those with 13 or more years of schooling). It’s worth noting that the same categorization is employed by the NFHS in its national reports.
Social groups	Social groups have been categorized into four distinct groups in accordance with the official classification of the Government of India: ‘Scheduled Caste’ (SC) (coded as 1): These communities/castes, often referred to as Dalits, have historically experienced oppression and marginalization in India. ‘Scheduled Tribe’ (ST) (coded as 2): This category encompasses indigenous and tribal communities who have historically faced social and economic disadvantages. ‘Other Backward Classes’ (OBC) (coded as 3): These include social groups/castes have faced social and educational disadvantages, but they may not be part of the SC or ST. ‘Others’ (coded as 4): This broad category includes all other communities and castes that do not fall under the SC, ST, or OBC classifications.
Religion	In the survey, women were asked to specify their religion, with response options including Hindu, Muslim, Christian, Sikh, Buddhist/Neo Buddhist, Jain, Jew, Parsi/Zoroastrian, Others, and no religion. Respondents were categorized into four distinct groups: ‘Hindu’ (coded as 1), ‘Muslim’ (coded as 2), ‘Christian’ (coded as 3), ‘Others’ (coded as 4). The ‘Others’ category encompasses individuals who identified as Sikh, Buddhist/Neo Buddhist, Jain, Jew, Parsi/Zoroastrian, specified ‘Others’, or indicated ‘no religion.‘
Household wealth	Household wealth status was evaluated using wealth scores, which were assigned to households through principal component analysis. These scores were calculated based on various indicators, including ownership of consumer goods and housing characteristics such as toilet facilities, flooring materials, and the source of drinking water. Subsequently, households were ranked according to their respective scores and divided into five equal groups, known as quintiles. These are also known as household wealth quintiles. The breakdown is as follows: The lowest 20% were classified as ‘poorest’ (coded as 1). The subsequent 20% were categorized as ‘poorer’ (coded as 2). The middle 20% were placed in the ‘middle’ category (coded as 3). The following 20% were labeled as ‘richer’ (coded as 4). The top 20% were designated as the ‘richest’ (coded as 5). It’s crucial to emphasize that the authors did not generate this variable themselves; rather, they relied on the variable provided within the datasets. For a more comprehensive understanding of this variable, readers are encouraged to consult the national reports of the NFHS.
Regions	Indian states have been regrouped into six regions. ‘North’ region (coded as 1) includes Himachal Pradesh, Punjab, Rajasthan, Haryana, Uttarakhand; ‘Central’ region (coded as 2) includes the states of Uttar Pradesh, Madhya Pradesh and Chhattisgarh; ‘East’ region (coded as 3) includes the states of Bihar, Jharkhand, West Bengal and Odisha; ‘West’ region (coded as 4) includes the states of Gujarat, Maharashtra and Goa; ‘South’ region (coded as 5) includes the states of Kerala, Karnataka, Andhra Pradesh and Tamil Nadu; ‘Northeast’ region (coded as 6) includes the states of Sikkim, Assam, Meghalaya, Manipur, Mizoram, Tripura, and Arunachal Pradesh.
***Health and behavioral variables***	
Currently having diabetes	In NFHS, women were asked, “Do you currently have diabetes?” with two response options: “No” (coded as 0) for those without diabetes and “Yes” (coded as 1) for those who reported having diabetes.
Anaemia level	This variable represents the level of anaemia, and it is categorized as an ordinal variable with four distinct groups: ‘Severe anaemia’ (coded as 1): This category is defined as hemoglobin levels of 8.0 g/dl or less for non-pregnant women and 7.0 g/dl or less for pregnant women. ‘Moderate anaemia’ (coded as 2): Moderate anaemia encompasses hemoglobin levels ranging from 8.0 to 10.9 g/dl for non-pregnant women and 7.0 to 9.9 g/dl for pregnant women. ‘Mild anaemia’ (coded as 3): For non-pregnant women, mild anaemia is characterized by hemoglobin levels falling between 11.0 to 11.9 g/dl, while for pregnant women, it corresponds to hemoglobin levels between 10.0 to 10.9 g/dl. ‘No anaemia’ (coded as 4): non-pregnant women with hemoglobin levels exceeding 11.0 g/dl and pregnant women with hemoglobin levels surpassing 12.0 g/dl are categorized as having no anaemia.
Mass media exposure	In NFHS, women were asked three questions related to their mass media exposure: “How often do women read newspapers/magazines?”, “How often do women watch television?”, “How often do women listen to the radio?” For each of these questions, respondents could choose from the following response options: “almost every day”, “at least once a week”, “less than once a week”, and “not at all”. A new variable was created by combining these responses into two categories: ‘No exposure’ (coded as 0): This category included women who reported no exposure to any of the mass media types (i.e., they answered ‘not at all’ for all three questions). ‘Have exposure’ (coded as 1): Women who responded “almost every day”, “at least once a week”, or “less than once a week” for at least one type of mass media were classified under this category.
Current contraceptive use	In NFHS, women were asked whether they were doing something or using any method to delay or avoid getting pregnant. This question had 20 possible responses, they were: not using any method, or using pill, Intrauterine device (IUD), injection, diaphragm, male condom, female condom, male sterilization, female sterilization, periodic abstinence, withdrawal, other traditional, implants/Norplant, prolonged abstinence, lactational amenorrhea (LAM), foam or jelly, emergency contraception, other modern method, standard days method (SDM), specific method 1, specific method 2. These responses have been recoded into two categories: ‘no or traditional’ contraceptive user (coded as 0), if a woman did not use anything, or used periodic abstinence, withdrawal, other traditional method, or prolonged abstinence to delay or avoid getting pregnant; and ‘modern’ contraceptive user (coded as 1) if a woman used pill, IUD, injections, diaphragm, male condom, female sterilization, male sterilization, implants/Norplant, LAM, female condom, foam or jelly, emergency contraception, other modern method, SDM, specific method 1, or specific method 2.
Alcohol consumption	In NFHS, women were asked a question: “Do you drink alcohol?” The coding for this variable is as follows: ‘No’ (coded as 0): Women who responded ‘no’ to the question, indicating that they did not drink alcohol. ‘Yes’ (coded as 1): Women who responded ‘yes’ to the question, indicating that they drunk alcohol.
Consumption of tobacco in any form	In NFHS, women were asked about their habits related to smoking and tobacco consumption. The coding for this variable is as follows: ‘Uses tobacco: smoke or smokeless’ (coded as 1): Women who reported using any of the following tobacco products - cigarettes, pipes, chewing tobacco, snuff, cigars, *gutka/paan, paan* with tobacco, *hookah, khaini, or bidis* - were categorized as users of tobacco, whether in smoke or smokeless form. ‘No tobacco’ (coded as 0): Women who did not use any of the mentioned tobacco products were classified under this category, indicating that they did not use tobacco in any form.
Year	Three NFHS were conducted in three different time periods. They were coded as: ‘NFHS 3’, conducted during 2005-06 (coded as 0), ‘NFHS 4’, conducted during 2015-16 (coded as 1), and ‘NFHS 5’, conducted during 2019-21 (coded as 2).

### Statistical analysis

We used bivariate statistics to analyze the prevalence of overweight/obesity among WRA across various background characteristics. All estimates of overweight/obesity were appropriately weighted. We assessed the statistical significance of the association between each independent variable and the outcome variable using the Chi-squared test [40]. Moreover, our study employed multivariable binary logistic regression to quantify the independent effects of determinants of overweight/obesity [41].

We constructed three logistic regression models, each comprising a distinct set of independent variables. We utilized a block-wise forward selection method to eliminate any variables that were statistically insignificant (p > 0.05). Variables were introduced in blocks, and only those with a p < 0.05 were included in the subsequent models. Model 1 included biodemographic variables, such as age, marital status, and parity. Model 2 expanded upon Model 1 by incorporating statistically significant variables from it, as well as socioeconomic and geographic variables, such as education level, social groups, religion, household wealth, and region of residence. Finally, Model 3 extended the analysis further by including significant variables from Model 2, as well as health and behavior-related variables, such as diabetes, anaemia level, mass media exposure, current contraceptive use, and tobacco consumption. We reported Adjusted Odds Ratios (AOR), p-values (< 0.05), and 95% Confidence Intervals (CIs) to present the outcomes of the logistic regression models. To ensure that the independent variables did not suffer from multicollinearity issues, we calculated the variance inflation factors (VIF) (see Supplementary Table 1) [42]. We found that the VIFs for all the independent variables were below 5. This indicates that multicollinearity was not a concern for our models. We also took into consideration the complex design of NFHS surveys by using the *‘svyset’* command in Stata 16 statistical software [43].

## Results

### Respondent characteristics

Table 2 presents the socio-demographic profile of WRA in NFHS-3, NFHS-4, and NFHS-5. In each round of NFHS, about one-third and two-thirds of women were aged 20–29 and currently married, respectively. Regarding parity, more than one-third of women in NFHS-3 had 1–2 children, while this was close to half of women in NFHS-4 and NFHS-5. Half of the women had a secondary education, and only a tiny percentage of women belonged to Scheduled Tribe (ST) social group. Nearly 80% and 50% of women were Hindu and belonged to the richest quintile across all rounds of NFHS, respectively. The proportion of women was higher in the southern region of the country. More than 90% and 50% of women had exposure to mass media and use modern contraceptives in all rounds of NFHS, respectively. Most women in all rounds of NFHS did not use tobacco or drunk alcohol.

**Table 2:** Respondent characteristics

Background characteristics	NFHS-3 (2005-06)		NFHS-4 (2015-16)		NFHS-5 (2019-21)	
	Frequency (N = 44,882)	%	Frequency (N = 171,443)	%	Frequency (N = 135,272)	%
***Biodemographic variables***						
**Age (in years)**						
15–19	8,238	18.36	26,226	15.30	19,787	14.63
20–29	15,616	34.79	58,132	33.91	42,996	31.79
30–39	12,435	27.71	47,661	27.80	39,271	29.03
40–49	8,593	19.15	39,424	23.00	33,217	24.56
**Marital status**						
Currently married	31,644	70.51	121,244	70.72	94,916	70.17
Not married	11,159	24.86	42,353	24.70	33,965	25.11
Formerly married	2,079	4.63	7,846	4.58	6,390	4.72
**Parity**						
No children	14,440	32.17	55,298	32.25	43,204	31.94
1–2 children	16,118	35.91	76,060	44.36	62,883	46.49
3–4 children	10,358	23.08	32,629	19.03	24,462	18.08
5 and above	3,966	8.84	7,455	4.35	4,724	3.49
***Socioeconomic and geographic variables***						
**Level of education**						
No education	9,809	21.86	26,685	15.56	17,310	12.80
Primary	5,647	12.58	17,359	10.13	12,360	9.14
Secondary	22,450	50.02	88,530	51.64	70,835	52.36
Higher	6,976	15.54	38,869	22.67	34,768	25.70
**Social groups**						
SC	7,847	17.48	30,513	17.80	28,024	20.72
ST	1,257	2.80	7,447	4.34	6,184	4.57
OBC	17,375	38.71	80,660	47.05	63,742	47.12
Others	18,403	41.00	52,823	30.81	37,323	27.59
**Religion**						
Hindu	35,179	78.38	131,499	76.70	107,343	79.35
Muslim	6,705	14.94	29,113	16.98	20,784	15.36
Christian	1,365	3.04	4,554	2.66	3,631	2.68
Others	1,634	3.64	6,276	3.66	3,515	2.60
**Household wealth**						
Poorest	1,131	2.52	4,727	2.76	4,095	3.03
Poorer	2,677	5.96	11,404	6.65	9,869	7.30
Middle	6,080	13.55	26,673	15.56	22,228	16.43
Richer	12,931	28.81	53,314	31.10	41,245	30.49
Richest	22,063	49.16	75,325	43.94	57,835	42.75
**Regions**						
North	5,120	11.41	19,404	11.32	15,186	11.23
Central	8,500	18.94	36,140	21.08	28,787	21.28
East	7,417	16.53	23,761	13.86	22,097	16.34
Northeast	1,060	2.36	3,182	1.86	2,602	1.92
West	9,984	22.24	36,326	21.19	27,726	20.50
South	12,802	28.52	52,630	30.70	38,874	28.74
***Health and behavioral variables***						
**Currently having diabetes**						
No	44,235	98.56	167,068	97.45	131,744	97.39
Yes	647	1.44	4,375	2.55	3,528	2.61
**Anaemia level**						
Severe	702	1.56	1586	0.93	3197	2.36
Moderate	6165	13.74	19,761	11.53	35,470	26.22
Mild	16,071	35.81	65,562	38.24	34,094	25.20
Not anemic	21,945	48.89	84,534	49.31	62,511	46.21
**Mass media exposure**						
No exposure	3,032	6.76	9,288	5.42	11,918	8.81
Have exposure	41,850	93.24	162,155	94.58	123,354	91.19
**Current contraceptive use**						
No or traditional	26,151	58.27	105,211	61.37	76,092	56.25
Modern	18,731	41.73	66,232	38.63	59,180	43.75
**Alcohol consumption**						
No	44,633	99.44	170,296	99.33	134,806	99.66
Yes	249	0.56	1,147	0.67	466	0.34
**Consumption of tobacco in any form**						
No tobacco	41,793	93.12	164,291	95.83	131,806	97.44
Uses tobacco: smoke or smokeless	3,089	6.88	7,152	4.17	3,466	2.56

Note: N: sample size; SC: Scheduled Caste; ST: Scheduled Tribe; OBC: Other Backward Classes. All percentages are weighted

### Prevalence of overweight/obesity among WRA by background characteristics

In NFHS-3, approximately 23% of WRA in urban India were reported as overweight/obese. However, this percentage increased to 30% in NFHS-4 and further to 33% in NFHS-5. Table 3 presents the prevalence of overweight/obesity during 2005–2021 among WRA in urban India by background characteristics. Across all rounds of the NFHS, there is a consistent trend of rising overweight/obesity rates with advancing age. Notably, around half of women aged 40–49 exhibited overweight/obesity, while this was observed in less than 10% of women aged 15–19. Furthermore, a distinct pattern emerges regarding marital status and parity. Across all survey rounds, persistently elevated prevalence of overweight/obesity was observed among currently married women and women with children as compared to those not married and with no children.

**Table 3:** Prevalence of overweight/obesity among WRA by background characteristics in urban India during NFHS 3, 4 and 5

Background characteristics	NFHS 3		NFHS 4		NFHS 5	
	Prevalence of Overweight/Obesity (%)	95% CI [Lower, Upper]	Prevalence of Overweight/Obesity (%)	95% CI [Lower, Upper]	Prevalence of Overweight/Obesity (%)	95% CI [Lower, Upper]
**Biodemographic variables**						
**Age (in years)**	**χ**^**2**^ **= 4284.70, p-value: <0.001**		**χ**^**2**^ **= 17800.00, p-value: <0.001**		**χ**^**2**^ **= 12400.00, p-value: <0.001**	
15–19	4.96	[4.30,5.70]	7.51	[6.917,8.14]	8.751	[8.08,9.48]
20–29	15.22	[14.20,16.31]	20.93	[20.26,21.62]	23.01	[22.33,23.71]
30–39	31.61	[30.09,33.17]	39.90	[39.01,40.79]	41.80	[40.92,42.69]
40–49	41.78	[39.98,43.60]	49.77	[48.83,50.72]	48.76	[47.84,49.69]
**Marital status**	**χ**^**2**^ **= 1923.79, p-value: <0.001**		**χ**^**2**^ **= 10800.00, p-value: <0.001**		**χ**^**2**^ **= 8650.02, p-value: <0.001**	
Currently married	28.09	[26.90,29.32]	37.47	[36.83,38.11]	39.66	[38.98,40.35]
Not married	7.84	[6.98,8.81]	10.57	[9.996,11.17]	12.22	[11.64,12.83]
Formerly married	26.02	[23.37,28.87]	36.62	[34.62,38.67]	38.18	[36.40,40.00]
**Parity**	**χ**^**2**^ **= 1835.33, p-value: <0.001**		**χ**^**2**^ **= 10700.00, p-value: <0.001**		**χ**^**2**^ **= 8233.01, p-value: <0.001**	
No children	10.72	[9.801,11.71]	14.13	[13.53,14.75]	15.81	[15.24,16.41]
1–2 Children	29.37	[27.92,30.86]	38.09	[37.31,38.88]	40.67	[39.89,41.47]
3–4 Children	29.16	[27.51,30.86]	40.54	[39.56,41.53]	40.79	[39.78,41.82]
5 and above	25.34	[23.09,27.73]	37.03	[35.43,38.66]	39.19	[37.21,41.21]
**Socioeconomic and geographic variables**						
**Level of education**	**χ**^**2**^ **= 451.10, p-value: <0.001**		**χ**^**2**^ **= 85.46, p-value: <0.001**		**χ**^**2**^ **= 134.80, p-value: <0.001**	
No education	16.90	[15.58,18.31]	29.69	[28.71,30.68]	32.68	[31.57,33.81]
Primary	20.97	[19.27,22.77]	33.64	[32.35,34.96]	37.12	[35.76,38.51]
Secondary	23.76	[22.61,24.96]	30.49	[29.82,31.18]	31.83	[31.17,32.51]
Higher	30.54	[28.65,32.50]	30.91	[29.93,31.92]	32.92	[32.04,33.81]
**Social groups**	**χ**^**2**^ **= 542.89, p-value: <0.001**		**χ**^**2**^ **= 1013.70, p-value: <0.001**		**χ**^**2**^ **= 573.52, p-value: <0.001**	
SC	16.01	[14.56,17.59]	26.16	[25.02,27.34]	28.40	[27.42,29.39]
ST	11.19	[8.849,14.05]	21.42	[19.89,23.04]	24.94	[22.91,27.09]
OBC	22.06	[20.59,23.60]	30.76	[30.09,31.44]	33.66	[32.96,34.37]
Others	27.58	[26.25,28.96]	34.81	[33.89,35.74]	35.59	[34.57,36.63]
**Religion**	**χ**^**2**^ **= 36.74, p-value: 0.032**		**χ**^**2**^ **= 63.36, p-value: 0.002**		**χ**^**2**^ **= 112.60, p-value: <0.001**	
Hindu	22.63	[21.54,23.76]	30.53	[29.93,31.13]	32.48	[31.88,33.10]
Muslim	22.87	[20.65,25.25]	30.96	[29.83,32.11]	32.13	[30.76,33.52]
Christian	25.06	[21.94,28.46]	35.94	[33.57,38.38]	40.50	[38.11,42.93]
Others	28.77	[24.13,33.90]	31.60	[29.18,34.13]	34.79	[32.28,37.38]
**Household wealth**	**χ**^**2**^ **= 2361.81, p-value: <0.001**		**χ**^**2**^ **= 4644.30, p-value: <0.001**		**χ**^**2**^ **= 2650.35, p-value: <0.001**	
Poorest	4.40	[2.934,6.551]	9.11	[7.982,10.37]	13.52	[12.08,15.10]
Poorer	6.76	[5.28,8.62]	15.25	[13.92,16.68]	20.47	[19.07,21.94]
Middle	11.47	[10.19,12.89]	22.82	[21.92,23.74]	26.92	[26.02,27.85]
Richer	17.52	[16.34,18.76]	30.76	[30.02,31.52]	32.21	[31.44,32.99]
Richest	32.24	[31.04,33.47]	37.33	[36.58,38.08]	38.72	[37.89,39.57]
**Regions**	**χ**^**2**^ **= 345.40, p-value: <0.001**		**χ**^**2**^ **= 1699.35, p-value: <0.001**		**χ**^**2**^ **= 2233.31, p-value: <0.001**	
North	24.73	[21.96,27.72]	26.42	[25.49,27.36]	30.64	[29.43,31.89]
Central	18.61	[16.69,20.69]	25.64	[24.94,26.34]	28.47	[27.42,29.54]
East	19.07	[16.89,21.46]	27.11	[25.66,28.61]	27.76	[26.36,29.20]
Northeast	17.08	[15.05,19.33]	25.35	[23.94,26.81]	27.18	[25.51,28.91]
West	23.24	[21.21,25.41]	32.81	[31.43,34.21]	29.52	[28.07,31.01]
South	27.67	[25.66,29.78]	36.52	[35.48,37.57]	42.10	[41.14,43.07]
**Health and Behavioral variables**						
**Currently having diabetes**	**χ**^**2**^ **= 358.47, p-value: <0.001**		**χ**^**2**^ **= 1897.19, p-value: <0.001**		**χ**^**2**^ **= 1082.32, p-value: <0.001**	
No	22.51	[21.52,23.53]	30.00	[29.47,30.53]	32.02	[31.45,32.59]
Yes	54.05	[48.67,59.33]	60.79	[57.27,64.20]	58.35	[55.73,60.92]
**Anaemia level**	**χ**^**2**^ **= 408.03, p-value: <0.001**		**χ**^**2**^ **= 585.55, p-value: <0.001**		**χ**^**2**^ **= 322.48, p-value: <0.001**	
Severe	7.27	[4.97,10.53]	19.08	[15.62,23.11]	26.93	[24.67,29.32]
Moderate	16.00	[14.50,17.63]	26.64	[25.59,27.72]	30.15	[29.34,30.98]
Mild	21.88	[20.64,23.17]	29.14	[28.40,29.88]	31.66	[30.81,32.51]
Not anemic	26.21	[25.08,27.38]	33.25	[32.60,33.90]	35.02	[34.29,35.76]
**Mass Media Exposure**	**χ**^**2**^ **= 280.61, p-value: <0.001**		**χ**^**2**^ **= 470.88, p-value: <0.001**		**χ**^**2**^ **= 267.33, p-value: <0.001**	
No exposure	10.61	[9.09,12.35]	20.67	[19.29,22.13]	25.99	[24.75,27.28]
Have exposure	23.86	[22.86,24.89]	31.36	[30.82,31.91]	33.35	[32.76,33.95]
**Current contraceptive use**	**χ**^**2**^ **= 1097.01, p-value: <0.001**		**χ**^**2**^ **= 4173.15, p-value: <0.001**		**χ**^**2**^ **= 2863.37, p-value: <0.001**	
No or traditional	17.40	[16.37,18.48]	25.07	[24.51,25.64]	26.68	[26.08,27.30]
Modern	30.73	[29.40,32.10]	39.86	[39.01,40.71]	40.44	[39.64,41.26]
**Alcohol consumption**	**χ**^**2**^ **= 0.29, p-value: 0.655**		**χ**^**2**^ **= 0.592, p-value: 0.632**		**χ**^**2**^ **= 3.49, p-value: 0.149**	
No	22.96	[21.96,23.98]	30.79	[30.25,31.33]	32.72	[32.15,33.29]
Yes	24.40	[18.47,31.51]	29.74	[25.67,34.15]	28.65	[23.62,34.27]
**Consumption of tobacco in any form**	**χ**^**2**^ **= 38.90, p-value: <0.001**		**χ**^**2**^ **= 40.68, p-value: <0.001**		**χ**^**2**^ **= 21.39, p-value: <0.001**	
No tobacco	23.30	[22.29,24.34]	30.93	[30.39,31.47]	32.80	[32.22,33.38]
Uses tobacco: smoke or smokeless	18.41	[16.20,20.85]	27.37	[25.68,29.14]	29.07	[27.10,31.12]
**Prevalence of overweight/obesity among WRA in urban India**	**22.96**	[21.97,23.99]	**30.78**	[30.25,31.32]	**32.70**	[32.13,33.28]

Note: Chi-squared test applied for each variable, CI: Confidence interval, WRA: Women of reproductive age. All percentages are weighted.

Over time, there has been a reduction in the disparities in overweight/obesity rates among different educational groups. In 2019-21, these differences have narrowed significantly, ranging from 32 to 37%, compared to larger gaps observed in 2005-06 (17–31%). This shift can be attributed to a faster increase in overweight/obesity rates among those with lower education levels (rising from 17 to 33% for those with no formal education and from 21 to 37% for those educated up to the primary level) compared to individuals with higher education (who experienced a more modest increase from 31 to 33%).

The prevalence of overweight/obesity also varied among different social groups. Specifically, the rates were relatively lower among Scheduled Caste (SC) and ST women in comparison to Other Backward Class (OBC) and ‘Other’ category women. However, the rate of increase in overweight/obesity prevalence was more pronounced among SC and ST women. The prevalence of overweight/obesity demonstrated a consistent upward trend as household wealth increased. This pattern was observed consistently across all rounds of the NFHS. Particularly noteworthy was the significantly higher prevalence among the wealthiest segment of the population, the richest quintile, displaying rates of 32%, 37%, and 39% in consecutive survey rounds. However, the most substantial increase 14 to 15 percentage points (pp) was witnessed among the three quintiles in the middle of the wealth distribution (i.e. poorer, middle, and richer).

In addition, women with mass media exposure and those utilizing traditional contraception methods consistently displayed higher levels of overweight/obesity throughout all survey rounds. Overweight/obesity prevalence was notably higher among women with diabetes compared to those without diabetes. In the period of 2019-21, the southern region of India recorded a notably higher prevalence of overweight/obesity at 42%, in contrast to approximately 25–30% prevalence in other regions across the country. Remarkably, the southern region also exhibited the most pronounced shift in prevalence over the study period of about 14 pp (28–42%).

### Prevalence of overweight/obesity among WRA across Indian states during 2005-2021

**Fig. 3:**
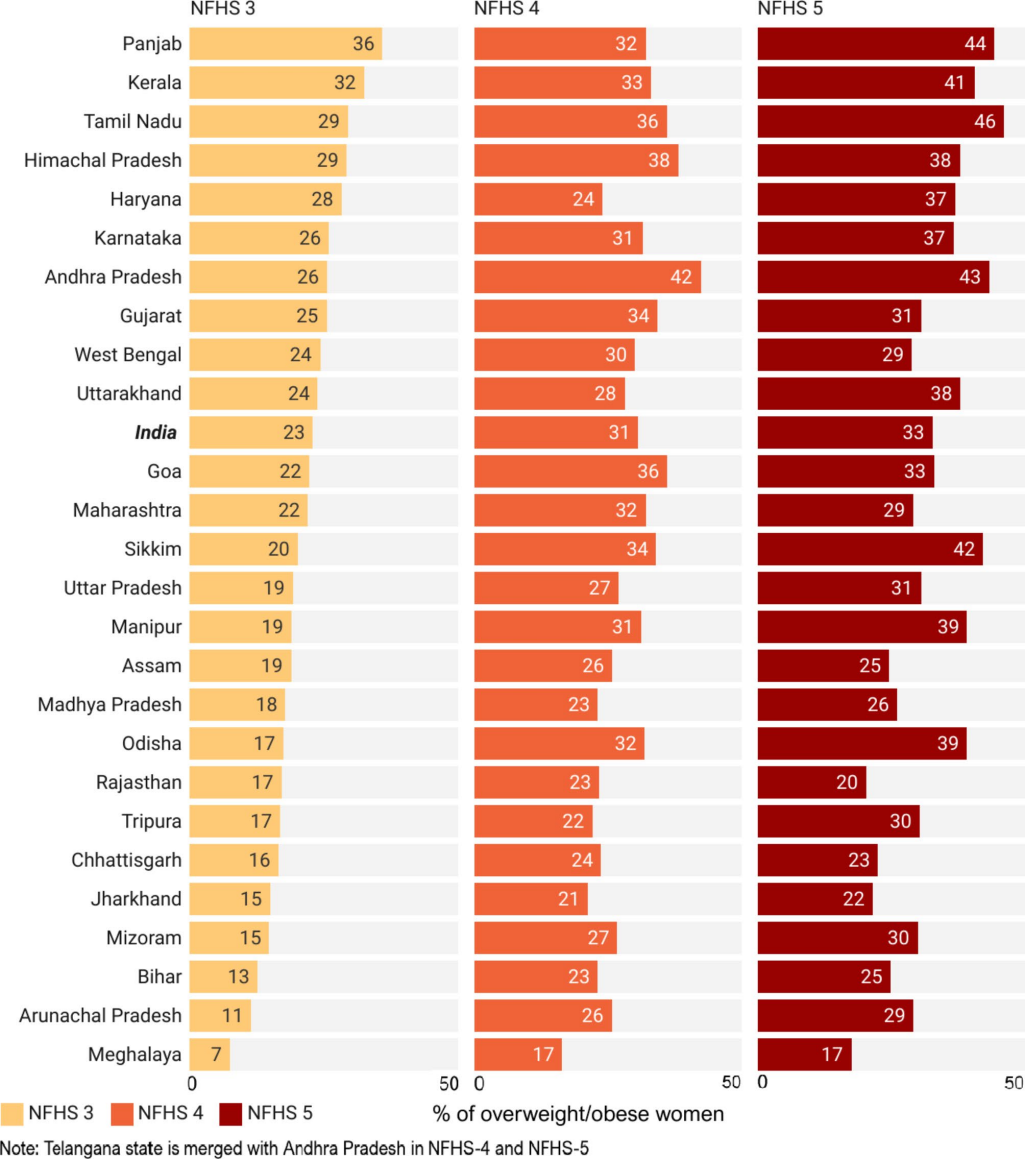
State-level disparities in the prevalence of overweight/obesity among WRA in urban India during NFHS-3, NFHS-4, and NFHS-5

There was a notable 10 pp increase in the prevalence of overweight/obesity among WRA in urban India between NFHS-3 (23%) and NFHS-5 (33%) (see Fig. 3). However, there were significant state-level disparities in the prevalence of overweight/obesity among WRA of urban India during three rounds of NFHS. However, it is important to discuss the spatial patterns observed in the most recent survey (2019-21). Two regions demonstrated particularly high levels of overweight/obesity: the southern region, which included Tamil Nadu (46%), Andhra Pradesh (43%), Kerala (41%), and Karnataka (37%), and the northwestern region comprising Punjab (44%), Himachal Pradesh (38%), Uttarakhand (38%), and Haryana (37%). In addition to these regions, other states with a prevalence exceeding 35% included Sikkim, Odisha, and Manipur. Conversely, most of the Empowered Action Group states, such as Bihar, Rajasthan, Madhya Pradesh, Chhattisgarh, Jharkhand, and Assam, reported relatively lower prevalence rates, typically around 25% or less.

### Spatiotemporal variation in overweight/obesity among WRA in urban India

**Fig. 4:**
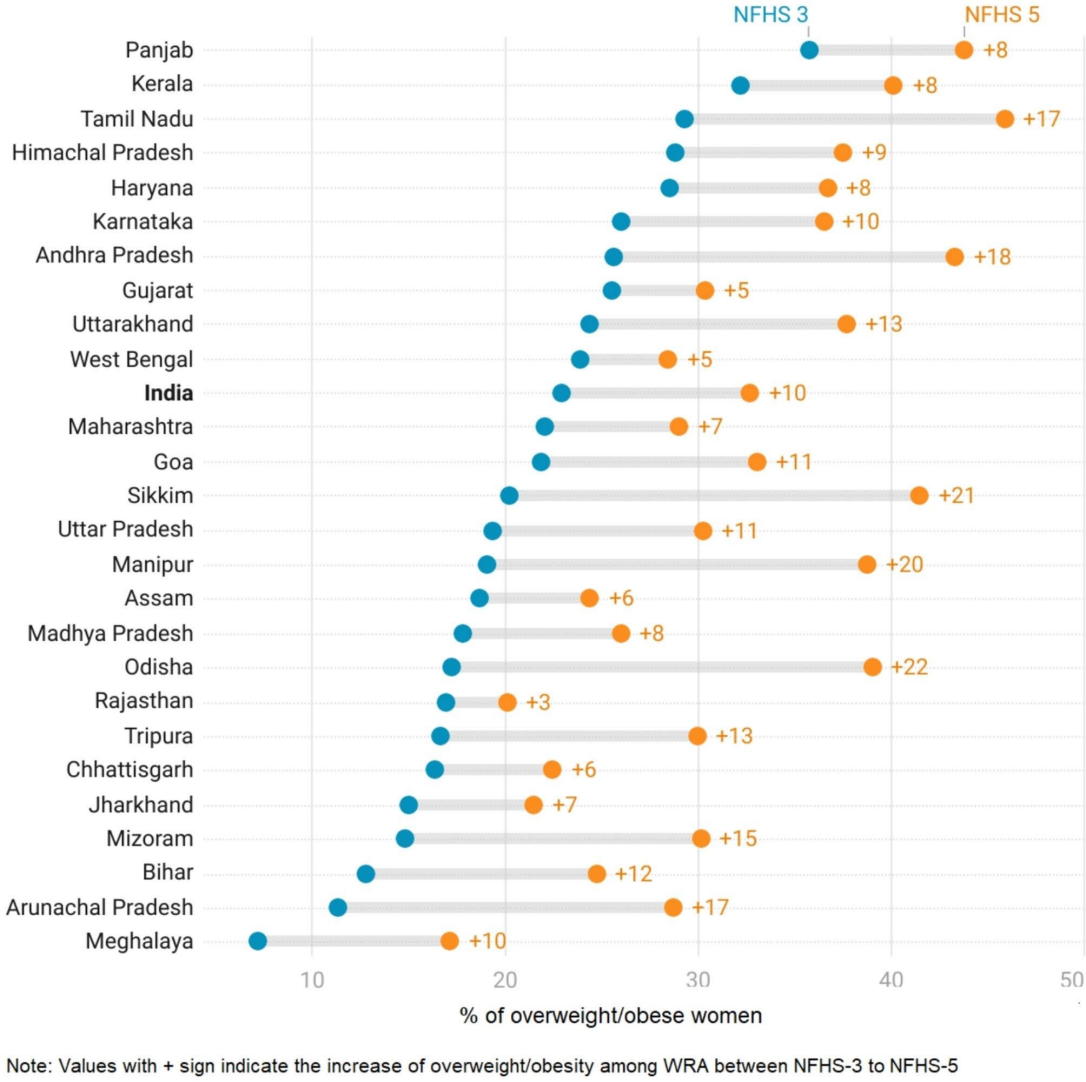
State-wise increase in overweight/obesity among WRA in urban India during 2005-2021

Figure 4 illustrates state wise increase in overweight/obesity among WRA in urban India between NFHS-3 (2005-06) and NFHS-5 (2019-21). Within the spectrum of all states, Odisha emerged with the most prominent increase in overweight/obesity prevalence, registering a substantial 22 pp increase. Notably, Sikkim (21 pp) and Manipur (20 pp) closely trailed behind as states with significant increments. States in southern and northeastern India witnessed a relatively higher increase in prevalence over the study period. For instance, in the South, Tamil Nadu and Andhra Pradesh experienced a 17 to 18 pp increase. Among the seven northeastern states, Sikkim (21 pp), Arunachal Pradesh, Manipur (20 pp), and Mizoram witnessed an increase of 15 pp or more. Importantly, none of the states demonstrated a decrease in overweight/obesity prevalence during the course of the study period. In contrast, Rajasthan had the lowest increase in overweight/obesity by three pp, followed by Gujarat (5 pp), West Bengal (5 pp), Assam (6 pp), and Chhattisgarh (6 pp).

### Determinants of overweight/obesity among WRA in urban India

Table 4 presents the adjusted odds ratios (AOR) for overweight/obesity among WRA in urban India. The final regression model (model 3) shows that the odds of overweight/obesity among women aged 40–49 years were were six times higher (AOR: 5.98, 95% CI: 5.71–6.27) than those aged 15–19. Likewise, the odds among currently married women were 1.86 times higher (AOR: 1.86, 95% CI: 1.79–1.93) than unmarried women. ST women had about 34% (AOR: 0.66, 95% CI: 0.63–0.68) lower odds than ‘Other’ category women. The odds among women with secondary education were 24% higher (AOR: 1.24, 95% CI: 1.21–1.28) than those with no education. In addition, women from the richest quintile had more than four times higher odds of being overweight/obese (AOR: 4.23, 95% CI: 3.95–4.54) compared to those from the poorest quintile. Women residing in the south were 1.77 times more (AOR: 1.77, 95% CI: 1.72–1.82) likely to be overweight/obese than those in the northern region. The odds of being overweight/obese were 1.92 times higher among women with diabetes (AOR: 1.92, 95% CI: 1.83–2.02). On the other hand, non-anemic women were 1.98 times more likely to be overweight/obese than women with severe anaemia.

**Table 4:** Adjusted odds ratios (with 95% CI) of overweight/obesity among WRA (15–49 years) in urban India, 2005-2021

Background characteristics	Model 1				Model 2				Model 3			
		95% CI				95% CI				95% CI		
	AOR	Lower	Upper	*P-*value	AOR	Lower	Upper	*P-*value	AOR	Lower	Upper	*P-*value
**Biodemographic variables**												
**Age (in years)**												
15–19®												
20–29	2.21	2.13	2.30	< 0.001	2.05	1.97	2.14	< 0.001	2.04	1.96	2.13	< 0.001
30–39	5.02	4.81	5.24	< 0.001	4.40	4.21	4.60	< 0.001	4.31	4.12	4.51	< 0.001
40–49	7.68	7.35	8.03	< 0.001	6.42	6.13	6.72	< 0.001	5.98	5.71	6.27	< 0.001
**Marital status**												
Not married®												
Currently married	1.75	1.68	1.82	< 0.001	1.85	1.78	1.92	< 0.001	1.86	1.79	1.93	< 0.001
Formerly married	1.27	1.20	1.33	< 0.001	1.57	1.49	1.65	< 0.001	1.57	1.49	1.66	< 0.001
**Parity**												
No children®												
1–2 children	1.11	1.08	1.15	< 0.001	1.12	1.08	1.15	< 0.001	1.17	1.13	1.21	< 0.001
3–4 children	0.91	0.88	0.95	< 0.001	1.16	1.12	1.21	< 0.001	1.28	1.23	1.33	< 0.001
5 and above	0.67	0.64	0.70	< 0.001	1.11	1.06	1.16	< 0.001	1.27	1.21	1.33	< 0.001
**Socioeconomic and geographic variables**												
**Level of education**												
No education®												
Primary					1.22	1.19	1.26	< 0.001	1.20	1.16	1.24	< 0.001
Secondary					1.33	1.29	1.36	< 0.001	1.24	1.21	1.28	< 0.001
Higher					1.37	1.33	1.41	< 0.001	1.23	1.19	1.27	< 0.001
**Social groups**												
Others®												
SC					0.83	0.81	0.86	< 0.001	0.80	0.78	0.82	< 0.001
ST					0.70	0.67	0.73	< 0.001	0.66	0.63	0.68	< 0.001
OBC					0.86	0.84	0.88	< 0.001	0.82	0.80	0.84	< 0.001
**Religion**												
Hindu®												
Muslim					1.22	1.19	1.25	< 0.001	1.18	1.15	1.21	< 0.001
Christian					1.03	0.98	1.07	0.229	1.03	0.98	1.07	0.249
Others					1.23	1.18	1.28	< 0.001	1.24	1.19	1.28	< 0.001
**Household wealth**												
Poorest®												
Poorer					1.53	1.42	1.65	< 0.001	1.50	1.39	1.61	< 0.001
Middle					2.28	2.13	2.44	< 0.001	2.23	2.08	2.39	< 0.001
Richer					3.04	2.84	3.25	< 0.001	3.00	2.80	3.21	< 0.001
Richest					4.18	3.91	4.48	< 0.001	4.23	3.95	4.54	< 0.001
**Regions**												
North®												
Central					1.04	1.02	1.07	0.002	1.06	1.03	1.09	< 0.001
East					1.16	1.12	1.20	< 0.001	1.22	1.18	1.25	< 0.001
Northeast					1.19	1.15	1.24	< 0.001	1.27	1.22	1.32	< 0.001
West					0.99	0.96	1.02	0.409	1.03	1.00	1.06	0.049
South					1.71	1.67	1.76	< 0.001	1.77	1.72	1.82	< 0.001
**Health and Behavioral variables**												
**Currently having diabetes**												
No®												
Yes									1.92	1.83	2.02	< 0.001
**Anaemia level**												
Severe®												
Moderate									1.53	1.42	1.65	< 0.001
Mild									1.67	1.55	1.80	< 0.001
Not anaemic									1.98	1.84	2.13	< 0.001
**Mass Media Exposure**												
No exposure®												
Have exposure									1.13	1.09	1.17	< 0.001
**Current contraceptive use**												
No or traditional®												
Modern									0.90	0.88	0.92	< 0.001
**Consumption of tobacco in any form**												
No tobacco®												
Uses tobacco: smoke or smokeless									0.86	0.83	0.89	< 0.001
**Year**												
NFHS 3®												
NFHS 4									1.44	1.40	1.48	< 0.001
NFHS 5									1.69	1.64	1.73	< 0.001

Notes: AOR: Adjusted odds ratios, CI: Confidence interval, ®: Reference category

## Discussion

Our study examined spatiotemporal variations and determinants of overweight/obesity among WRA living in urban areas of India during 2005-2021. Over time the prevalence of overweight/obesity among WRA in urban India has increased in all states, with substantial variation in magnitude of increase. Certain demographic groups, mainly, women with lower levels of education, women of SC/ST category, women from the three middle wealth quintiles (poorer, middle, and richer), and those living in the southern region have witnessed a more rapid increase in the overweight/obesity than remaining women. After controlling for a number of factors, the regression results revealed that being older, currently married, high parity, rich, higher educated, ‘Others’ social group,  region of residence, having diabetes and mass media exposure were important determinants of overweight/obesity among the WRA in urban India.

The increasing prevalence of overweight/obesity among WRA in urban India has become one of the most significant public health challenges facing the nation today [44]. However, over the past decade and a half, the growth of overweight/obesity has exhibited notably higher rates in some states along the eastern and northeastern regions in comparison to the rest of the country. The reasons behind this divergence remain unclear, necessitating further research to comprehend the factors contributing to the variable rates of overweight/obesity increase across different states. This finding underscores the imperative to incorporate geographical disparities in the rate of change in overweight/obesity among WRA in urban India into future policy frameworks designed to mitigate these elevated levels.

An important finding of this study is that overweight/obesity has surged at a notably higher rate among socioeconomically disadvantaged groups, specifically SC/ST women, and those with lower level of education. This observation contradicts the conventional expectation that overweight/obesity tend to increase more rapidly among the more affluent and educated segments of society [7, 8, 38, 45–47]. The factors driving this rapid rise in overweight/obesity within these vulnerable groups remain unclear. Consequently, further investigations are needed to shed light on this issue to guide future policy interventions.

The study reveals that age, parity, and marital status are significant biodemographic determinants of overweight/obesity among the WRA in urban India. The observation that older WRA are more likely to be overweight/obese aligns with findings from prior research conducted in Low and Middle-Income Countries, including India, Ethiopia, Zimbabwe, and China [6, 13, 48–51]. This increased risk may be attributed to several intertwined factors, including reduced physical activity, higher consumption of calorie-dense foods, the demands of child-rearing associated with advancing age, and age-related hormonal fluctuations [52–57]. The study also notes that married WRA were more likely to have overweight/obesity, a pattern consistent with previous research in countries like India, Maldives, Cambodia, and Greece [47, 58–60]. The increased likelihood of overweight/obesity among married WRA can be attributed to a mix of factors. These include gestational weight gain during pregnancy, the social expectations related to marriage that may encourage more frequent and calorie-rich meals, possibly resulting in higher calorie intake, and a reduced emphasis on monitoring body weight [38, 47]. A clear association between parity and overweight/obesity is also observed, which is consistent with similar results from studies conducted in China, the Maldives, and Iraq [53, 58, 61]. The relationship between the two is complex and can be influenced by various factors, including gestational weight gain, hormonal changes during pregnancy, and postpartum lifestyle adjustments [53, 58].

The study reveals that three significant socioeconomic factors, namely wealth, education, social group, are linked to overweight/obesity among WRA in urban India. WRA with higher education and from wealthier households are more likely to be overweight/obese than their less educated and poorer counterparts. These findings are consistent with previous research works in the countries of global South, including India, Bangladesh, Sub-Saharan Africa, Zimbabwe, Saudi Arabia, and China [15, 31, 45–47, 51, 62–64]. Evidences indicate that well-educated and wealthier WRA in urban areas often adopt sedentary lifestyles, engage in less physically demanding occupations, consume energy-dense foods due to their greater purchasing power, spend more time sitting rather than being active, and rely on modern conveniences such as smartphones [38, 47, 49, 65]. These lifestyle factors collectively contribute to an elevated risk of overweight/obesity among women in urban India [38, 47, 64]. In our study, ST women were less likely to be overweight/obese compared to women of other social groups, a trend consistent with prior Indian research [38, 47, 50, 64, 66]. This phenomenon may be attributed to a combination of factors prevalence among ST women, including economic disadvantages, traditional dietary patterns that are less calorie-dense, higher levels of physical activity due to manual labor or agricultural practices, cultural norms promoting healthier lifestyles, and potential genetic variations [38, 64, 66].

The results indicate that women from the southern region face a greater risk of overweight/obesity. This observation is consistent with findings reported in prior studies [6, 35, 47, 50, 66–68]. It is worth noting that these states are generally more developed, affluent, and further along in demographic and epidemiological transitions compared to other Indian [8, 69, 70]. In this study, anaemia and diabetes are significant predictors of overweight/obesity among WRA in urban India. Similar findings have been reported in earlier studies conducted in India [71–73]. It is important to note here that the relationship between these health conditions and overweight/obesity is complex. Therefore, additional research is necessary to delve deeper into these intricate connections.

A number of efforts have been made in the past in India to reduce or control the rising prevalence of overweight/obesity. The National Action Plan Monitoring Framework for Prevention and Control of NCDs was developed in 2013, aiming to halt the rise in obesity and diabetes prevalence in India by 2025 [74]. In 2017, the Ministry of Health and Family Welfare brought in a National Multisectoral Action Plan for Prevention and Control of Common NCDs and reiterated the same target [75]. Additionally, the National Nutrition Mission (*Poshan Abhiyaan*) aims to reduce the prevalence of stunting, malnutrition, and overweight/obesity among women and children [76]. Furthermore, the government has recently initiated several programs to promote physical activity, including the Fit India Movement [77], which encourages citizens to adopt an active lifestyle. In addition, some states at their state level implemented campaigns and programs to reduce overweight/obesity prevalence. For instance, in 2016, Tamil Nadu introduced both the Amma Master Health Care Scheme, and Maharashtra initiated an anti-obesity campaign with a focus on fostering healthy lifestyles and preventing overweight/obesity [78, 79]. However, despite the multitude of programs and policies currently in place, the concerning surge in overweight/obesity rates among WRA in urban India remains unabated. Unfortunately, the country is far from meeting the 2025 overweight/obesity targets set by the WHO [80]. This stark reality underscores the urgent need for a comprehensive review and reformulation of existing strategies aimed at reducing overweight/obesity prevalence in the country.

This study has certain limitations. This study employed a cross-sectional design, which permits the establishment of associations between dependent and independent variables. However, it’s important to note that this design does not enable us to infer causality. The models employed in this study were built exclusively using variables accessible in the survey data. Notably, we could not include in the analysis certain factors such as dietary behaviors, physical activity, and sleep patterns that may affect overweight/obesity as they were unavailable in the NFHS datasets. This may have potentially led to omitted variable bias. In light of these limitations, future research should prioritize the collection of more extensive and detailed data to enhance our understanding of the multifaceted factors influencing overweight/obesity.

## Conclusion

In conclusion, our study of spatiotemporal variations and determinants of overweight/obesity among WRA in urban India during 2005-2021 reveals a disconcerting trend of increasing prevalence, albeit with significant regional and demographic disparities. Rapid rise of overweight/obesity in some vulnerable groups of WRA and geographic regions, including less educated women, those from marginalized castes/social groups, those from the middle wealth quintiles, and residents of the southern region underscores the pressing need for targeted interventions and policies that address the specific challenges and disparities faced by these women. Future policies and interventions must prioritize these disadvantaged groups while promoting awareness and healthier lifestyles across WRA living in urban areas. A multifaceted approach is imperative to curbing the overweight/obesity epidemic and ensuring the well-being of India’s urban WRA population.

### Electronic supplementary material

Below is the link to the electronic supplementary material.

Supplementary Material 1

## Data Availability

National Family Health Survey datasets used in the study are available at the official website of Demographic and Health Surveys (DHS): https://dhsprogram.com/data/availabledatasets.cfm. In addition, these datasets can be obtained by registering as a DHS data user and requesting access for legitimate research purposes: https://dhsprogram.com/data/Access-Instructions.cfm.
