# Supplementary material for: Spatiotemporal variations and determinants of overweight/obesity among women of reproductive age in urban India during 2005-2021
Source: BMC Public Health. 2023 Oct 5;23:1933. doi: 10.1186/s12889-023-16842-x (PMC10557305; doi:10.1186/s12889-023-16842-x)
Supplement: Supplementary file 1 — Supplementary Material 1 [file 12889_2023_16842_MOESM1_ESM.docx]

**Supplementary Table 1: Variance inflation factors**

| **Background characteristics** | **VIF** | **1/VIF** |
| --- | --- | --- |
| **Biodemographic variables** |  |  |
| **Age (in years)** |  |  |
| 15-19® |  |  |
| 20-29 | 3.19 | 0.31 |
| 30-39 | 4.18 | 0.24 |
| 40-49 | 4.06 | 0.25 |
| **Marital status** |  |  |
| Not married® |  |  |
| Currently married | 4.46 | 0.22 |
| Formerly married | 1.86 | 0.54 |
| **Parity** |  |  |
| No children® |  |  |
| 1-2 children | 4.51 | 0.22 |
| 3-4 children | 3.91 | 0.26 |
| 5 and above | 2.04 | 0.49 |
| **Socioeconomic and geographic variables** |  |  |
| **Level of education** |  |  |
| No education® |  |  |
| Primary | 1.55 | 0.65 |
| Secondary | 2.89 | 0.35 |
| Higher | 2.97 | 0.34 |
| **Social groups** |  |  |
| Others® |  |  |
| SC | 1.50 | 0.67 |
| ST | 1.90 | 0.53 |
| OBC | 1.58 | 0.63 |
| **Religion** |  |  |
| Hindu® |  |  |
| Muslim | 1.15 | 0.87 |
| Christian | 1.71 | 0.58 |
| Others | 1.09 | 0.92 |
| **Household wealth** |  |  |
| Poorest® |  |  |
| Poorer | 1.26 | 0.79 |
| Middle | 1.33 | 0.75 |
| Richer | 1.41 | 0.71 |
| Richest | 1.37 | 0.73 |
| **Regions** |  |  |
| North® |  |  |
| Central | 2.01 | 0.50 |
| East | 1.69 | 0.59 |
| Northeast | 2.24 | 0.45 |
| West | 1.58 | 0.63 |
| South | 1.98 | 0.50 |
| **Health and Behavioral variables** |  |  |
| **Currently having diabetes** |  |  |
| No® |  |  |
| Yes | 1.03 | 0.97 |
| **Mass Media exposure** |  |  |
| No exposure® |  |  |
| Have exposure | 1.19 | 0.84 |
| **Current contraceptive use** |  |  |
| No or traditional® |  |  |
| Modern | 1.48 | 0.68 |
| **Consumption of tobacco in any form** |  |  |
| No tobacco® |  |  |
| Uses tobacco: smoke or smokeless | 1.23 | 0.81 |
| **Mean VIF** | 2.15 |  |

Note: ® Reference category.
